# Development and validation of a model to estimate the risk of acute ischemic stroke in geriatric patients with primary hypertension

**DOI:** 10.1186/s12877-021-02392-7

**Published:** 2021-08-09

**Authors:** Xifeng Zheng, Fang Fang, Weidong Nong, Dehui Feng, Yu Yang

**Affiliations:** 1 Department of Geriatrics, Affiliated Hospital of Guangdong Medical University, No.57, South of Renming Road, Zhanjiang, Guangdong 524001 People’s Republic of China; 2 Department of General Medicine, Huazhong University of Science and Technology Union Shenzhen Hospital, Shenzhen, Guangdong China; 3 Department of Neurology, Affiliated Minzu Hospital of Guangxi Medical University, Nanning, Guangxi China

**Keywords:** Acute ischemic stroke, Geriatric patients, Machine learning, Multivariable logistic regression, Primary hypertension

## Abstract

**Objectives:**

This study aimed to construct and validate a prediction model of acute ischemic stroke in geriatric patients with primary hypertension.

**Methods:**

This retrospective file review collected information on 1367 geriatric patients diagnosed with primary hypertension and with and without acute ischemic stroke between October 2018 and May 2020. The study cohort was randomly divided into a training set and a testing set at a ratio of 70 to 30%. A total of 15 clinical indicators were assessed using the chi-square test and then multivariable logistic regression analysis to develop the prediction model. We employed the area under the curve (AUC) and calibration curves to assess the performance of the model and a nomogram for visualization. Internal verification by bootstrap resampling (1000 times) and external verification with the independent testing set determined the accuracy of the model. Finally, this model was compared with four machine learning algorithms to identify the most effective method for predicting the risk of stroke.

**Results:**

The prediction model identified six variables (smoking, alcohol abuse, blood pressure management, stroke history, diabetes, and carotid artery stenosis). The AUC was 0.736 in the training set and 0.730 and 0.725 after resampling and in the external verification, respectively. The calibration curve illustrated a close overlap between the predicted and actual diagnosis of stroke in both the training set and testing validation. The multivariable logistic regression analysis and support vector machine with radial basis function kernel were the best models with an AUC of 0.710.

**Conclusion:**

The prediction model using multiple logistic regression analysis has considerable accuracy and can be visualized in a nomogram, which is convenient for its clinical application.

## Introduction

According to estimates by the World Health Organization, stroke is the second leading cause of death that will account for 7.8 million deaths and 23 million first-time ischemic stroke events by 2030 [1]. Many risk factors for stroke, such as hypertension, dyslipidemia, diabetes, smoking, and alcohol consumption, have been identified [2]. With rising levels of prosperity and an aging population, the prevalence of hypertension in China has increased from 23.4% in 1991 to 28.6% in 2011 (concerning approximately 300 million adults), which places a huge burden on public health resources [3]. Hypertensive patients commonly suffer acute ischemic strokes, especially among the elderly with multiple risk factors.

Considering the high fatality and disability rates resulting from stroke, we intended to develop a practical prediction model by integrating the common risk factors observed in the clinic. It is beneficial to estimate the risk of acute ischemic stroke in geriatric patients with primary hypertension so that appropriate preventive measures can be taken. Nomograms have been widely used for medical diagnosis and prognosis evaluation in recent years [4, 5] for their user-friendliness. Our aim was to provide an individualized clinical decision tool for physicians.

## Materials and methods

### Study design and data source

This retrospective file review entailed the extraction of information on geriatric patients who were older than 60 years [6] and diagnosed with primary hypertension, whether or not they suffered an acute ischemic stroke, from the electronic medical record database of the affiliated hospital of Guangdong medical university from October 2018 to May 2020. Patients with detailed clinical information, biochemical, and imaging examinations were included in the study. The diagnosis of acute ischemic stroke was based on neuroimaging.

This resulted in the files of a total of 1367 patients being analyzed in this retrospective study and randomly divided these into a training set and a testing set in a ratio of 70 to 30%.

### Study variables

A total of 15 risk factors associated with stroke were included in the study based on the literature [1, 7–9] and are listed in Table 1. Risk factors are indicators that can be easily assessed in clinical practice. All the risk factors were transformed into categorical variables to develop a nomogram. With this model, the sample size should be at least ten times greater than the number of variables [11].

**Table 1:** The risk factors with a definition in this study

Risk factors	Definition
Age (years)	Between 60 and 70 y, 70 and 80 y, and ≥ 80 y (the class interval included the lower value but excluded the higher one)
Sex	Female/Male
Degree of hypertension	Stage one (SBP = 140–160 mmHg and/or DBP = 90–100 mmHg) Stage two (SBP = 160–180 mmHg and/or DBP = 100–110 mmHg) Stage three (SBP > =180 mmHg and/or DBP > =110 mmHg)
Smoke	Yes/No (Based on the electronic medical record)
Alcohol abuse	Yes/No (Based on the electronic medical record)
Blood pressure management	Yes/No(Adhere to medication treatment and SBP < 140 mmHg [10])
Stroke history	Yes/No (Based on the electronic medical record)
Diabetes history	Yes/No (Based on the electronic medical record)
Coronary heart disease	Yes (Including paroxysmal or permanent atrial fibrillation)/No
Total cholesterol	Normal (3.1–5.7 mmol/L) Up-regulation (> 5.7 mmol/L)
Triglyceride	Normal (0.4–2.0 mmol/L) Up-regulation (> 2.0 mmol/L)
LDL- cholesterol	Normal (1.8–3.36 mmol/L) Up-regulation (> 3.36 mmol/L)
HDL- cholesterol	Normal (1.16–1.55 mmol/L) Up-regulation (> 1.55 mmol/L)
Homocysteine	Normal (6.5–16.9 μmol/L) Up-regulation (> 16.9 μmol/L)
Carotid artery stenosis	Yes/No (Based on the result of ultrasound)

### Statistical analysis

All variables were expressed as counts (%). Statistical analysis was performed using R software 3.6.1(http://www.R-project.org/). The risk factors showing a *P*-value < 0.05 in the Chi-square test were regarded as statistically significant. Multivariable logistic regression analysis was used to identify the optimal variables for the construction of the prediction model. These variables were expressed as odds ratios (ORs) with 95% confidence intervals (CIs) and *P*-values. The area under the curve (AUC) and calibration curves were used to assess the performance of the prediction model. A nomogram was developed to visualize the prediction model in a user-friendly manner [12, 13].

Furthermore, we applied four machine-learning classifiers (random forest, support vector machine with polynomial kernel, support vector machine with radial basis function kernel, and backpropagation neural network) using JupyterLab 1.2.6 (https://jupyterlab.readthedocs.io/en) to compare the results with the multivariable logistic regression model. The best combination of parameters of the machine learning algorithms was identified based on the highest log-likelihood. The average log-likelihood over five repetitions of fivefold cross-validation was used to select the optimal parameters [14].

## Results

### Baseline characteristics and optimal risk factors identification

Among the 1367 patients diagnosed with primary hypertension between October 2018 and May 2020 in this study, 437 had suffered an acute ischemic stroke. A total of 959 patients were assigned to the training set and 408 to the testing set. Detailed information about the characteristics of patients in the total cohort and the training set are shown in Tables 2 and Table 3, respectively.

**Table 2:** Baseline characteristics of the total cohort

Risk Factors	n(%)			
	Total = 1367	Stroke = 437	non-Stroke = 930	*P*-value
1.Age(years)				0.170
60y-70y	477(34.90)	139(31.81)	338(36.34)	
70y-80y	573(41.91)	198(45.31)	375(40.32)	
> =80y	317(23.19)	100(22.88)	217(23.34)	
2.Gender				0.021*
Female	592(43.30)	169(38.67)	423(45.48)	
Male	775(56.70)	268(61.33)	507(56.52)	
3.Degree of hypertension				0.071
Stage two	447(32.70)	158(36.16)	289(31.08)	
Stage three	920(67.30)	279(63.84)	641(68.92)	
4. Smoke				< 0.001*
Yes	105(7.68)	57(13.04)	48(5.16)	
No	1262(92.32)	380(86.96)	882(94.84)	
5. Alcohol abuse				< 0.001*
Yes	98(7.17)	54(12.36)	44(4.73)	
No	1269(92.83)	383(87.64)	886(95.27)	
6.Blood pressure management				< 0.001*
Yes	688(50.33)	128(29.30)	560(60.22)	
No	679(49.67)	309(70.70)	370(39.78)	
7. Stroke history				< 0.001*
Yes	280(20.48)	128(29.29)	152(16.34)	
No	1087(79.52)	309(70.71)	778(83.66)	
8. Diabetes history				< 0.001*
Yes	377(27.57)	147(33.64)	230(24.73)	
No	990(72.43)	290(66.36)	700(75.27)	
9. Coronary heart disease				0.775
Yes	302(22.10)	94(21.51)	208(22.37)	
No	1065(77.90)	343(78.49)	722(77.63)	
10. Total Cholesterol				< 0.001*
Normal	1125(82.30)	336(76.89)	789(84.84)	
UP-regulation	242(17.70)	101(23.11)	141(15.16)	
11. Triglyceride				0.180
Normal	1151(83.54)	356(81.46)	786(84.52)	
UP-regulation	225(16.46)	81(18.54)	144(15.48)	
12. LDL-C				< 0.001*
Normal	997(72.93)	289(66.13)	708(76.13)	
UP-regulation	370(27.07)	148(33.87)	222(23.87)	
13. HDL-C				0.251
Normal	1175(85.96)	383(87.64)	792(85.16)	
UP-regulation	192(14.04)	54(12.36)	138(14.84)	
14. Homocysteine				0.202
Normal	671(49.08)	226(51.72)	445(47.85)	
UP-regulation	696(50.92)	211(48.28)	485(52.15)	
15. Carotid artery stenosis				0.004*
Yes	78(5.71)	37(8.47)	41(4.41)	
No	1289(94.29)	400(91.53)	889(95.59)

**Table 3:** Baseline characteristics of the training set

Risk Factors	n(%)			
	Total = 959	Stroke = 309	non-Stroke = 650	*P*-value
1.Age(years)				0.389
60y-70y	325(33.89)	96(31.07)	229(35.23)	
70y-80y	405(42.23)	139(44.98)	266(40.92)	
> =80y	229(23.88)	74(23.95)	155(23.85)	
2.Gender				0.027*
Female	395(41.19)	111(35.92)	284(43.70)	
Male	564(58.81)	198(64.08)	366(56.30)	
3.Degree of hypertension				0.170
Stage two	314(32.74)	111(35.92)	203(31.23)	
Stage three	645(67.26)	198(64.08)	447(68.77)	
4. Smoke				< 0.001*
Yes	77(8.03)	44(14.24)	33(5.08)	
No	882(91.97)	265(85.76)	617(94.92)	
5. Alcohol abuse				< 0.001*
Yes	69(7.19)	40(12.95)	29(4.46)	
No	890(92.81)	269(87.05)	621(95.54)	
6.Blood pressure management				< 0.001*
Yes	488(50.89)	223(72.17)	265(40.77)	
No	471(49.11)	86(27.83)	385(59.23)	
7. Stroke history				< 0.001*
Yes	206(21.48)	91(29.45)	115(17.69)	
No	753(78.52)	218(70.55)	535(82.31)	
8. Diabetes history				< 0.001*
Yes	264(27.53)	101(32.69)	163(25.08)	
No	695(72.47)	208(67.31)	487(74.92)	
9. Coronary heart disease				0.454
Yes	211 (22.00)	63(20.39)	148(22.77)	
No	748(78.00)	246(79.61)	502(77.23)	
10. Total Cholesterol				0.005*
Normal	794(82.79)	240(77.67)	554(85.23)	
UP-regulation	165(17.21)	69(22.33)	96(14.77)	
11. Triglyceride				0.159
Normal	798(83.21)	249(80.58)	549(84.46)	
UP-regulation	161(16.79)	60(19.42)	101(15.54)	
12. LDL-C				0.003*
Normal	717(74.77)	212(68.61)	505(77.69)	
UP-regulation	242(25.23)	97(31.39)	145(22.31)	
13. HDL-C				0.765
Normal	835(87.07)	271(87.70)	564(86.77)	
UP-regulation	124(12.93)	38(12.30)	86(13.23)	
14. Homocysteine				0.224
Normal	477(49.74)	163(52.75)	314(48.31)	
UP-regulation	482(50.26)	146(47.25)	336(51.69)	
15. Carotid artery stenosis				0.004*
Yes	58(6.05)	29(9.39)	29(4.46)	
No	901(93.95)	280(90.61)	621(95.54)	

**Fig. 1:**
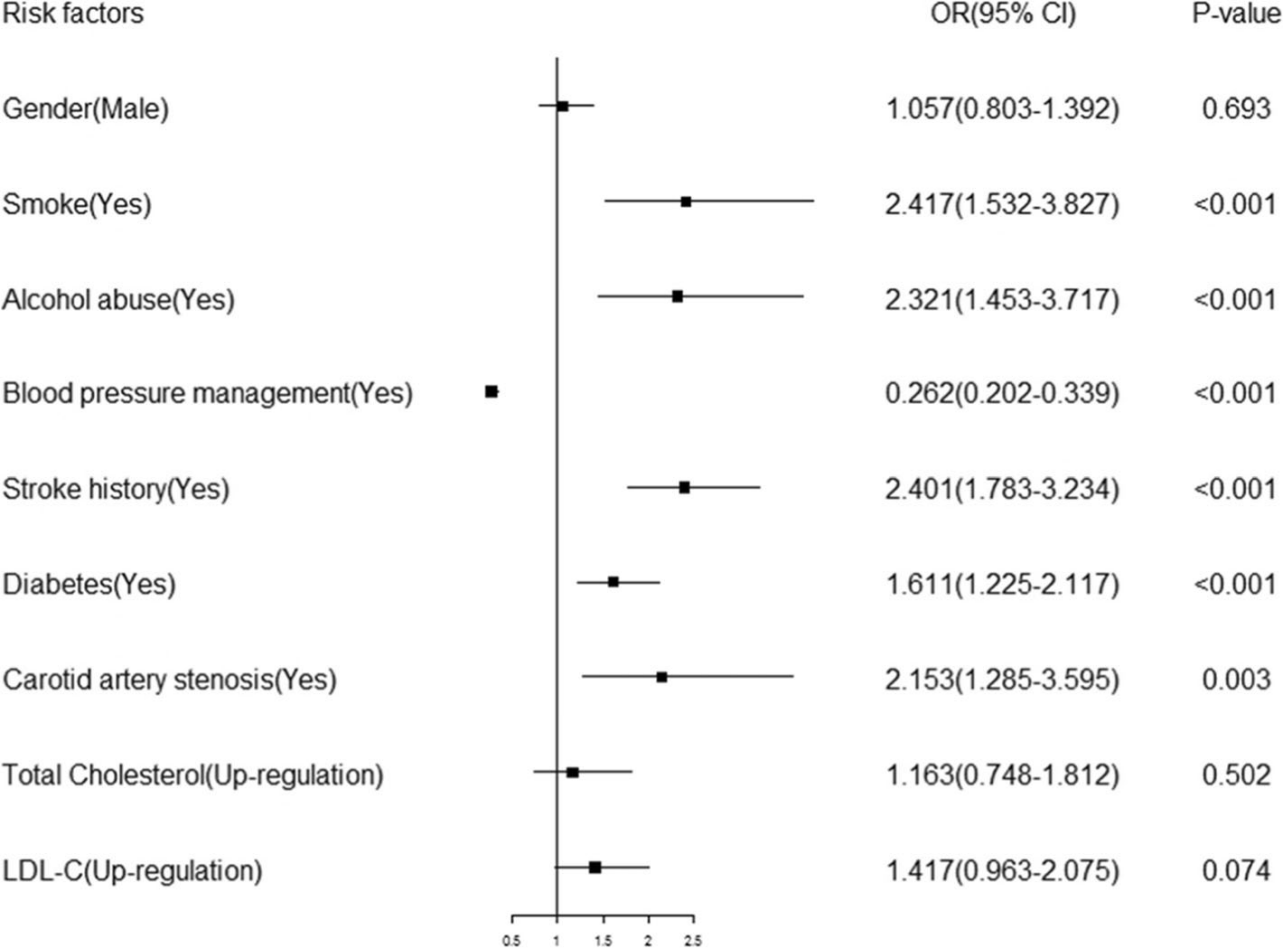
The risk factors in multivariable logistic regression analysis. Notes: OR = odds ratio, CI = confidence interval

There were nine variables (gender, smoking, alcohol abuse, blood pressure management, a history of stroke, diabetes, carotid artery stenosis (CAS), total cholesterol, and LDL-cholesterol) with statistically significant differences (*P* < 0.05) in the chi-square test. Six variables (smoking, alcohol abuse, blood pressure management, stroke history, diabetes, CAS) showed a statistically significant difference (*P* < 0.05) in the multivariable logistic regression analysis. The results of the multivariable logistic regression analysis are displayed as forest plots in Fig. 1.

### Construction and assessment of the prediction nomogram

**Fig. 2:**
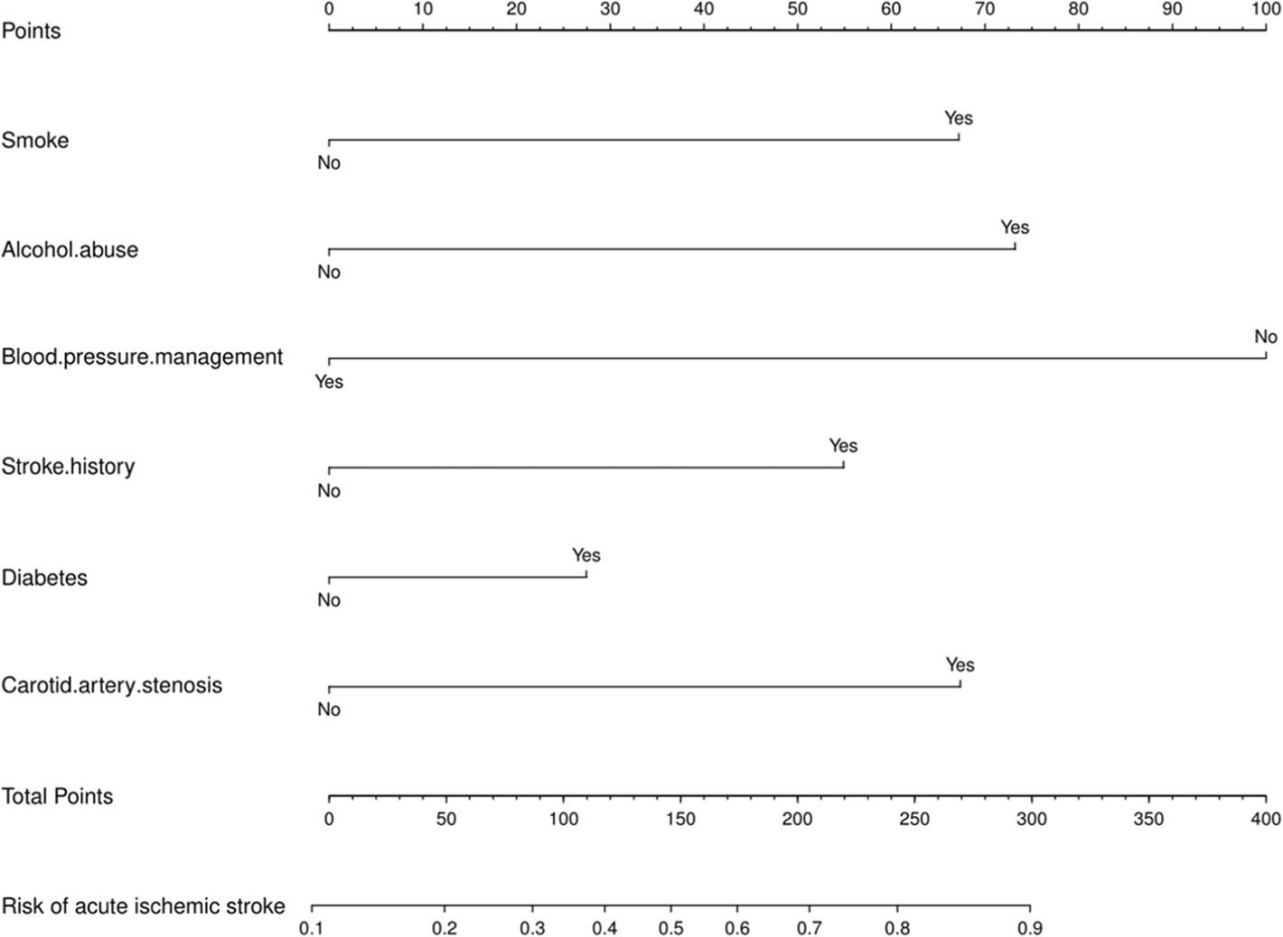
The nomogram for estimating risk of acute ischemic stroke

The prediction model was constructed by multivariable logistic regression based on the six identified variables (smoking, alcohol abuse, blood pressure management, stroke history, diabetes, CAS). The nomogram in Fig. 2 visualizes the model in a user-friendly manner.

Nomogram interpretation: The observed value of each feature variable was assigned a certain number of points by drawing a vertical line towards the top points scale. The sum of the points for each variable corresponded to the individual risk of acute ischemic stroke. If we assume that a geriatric patient has a history of ischemic stroke, smoking and poor blood pressure management, but no alcohol abuse or carotid stenosis, we can calculate the score of each feature of the patient according to the value of each variable: smoking (68 points) + history of ischemic stroke (54 points) + poor blood pressure management (100 points) + without alcohol abuse or carotid stenosis (0 points) =222 total points. From the total points scale, a line perpendicular to the acute ischemic risk scale at the bottom shows that the probability of acute ischemic stroke occurrence is about 75%.

**Fig. 3:**
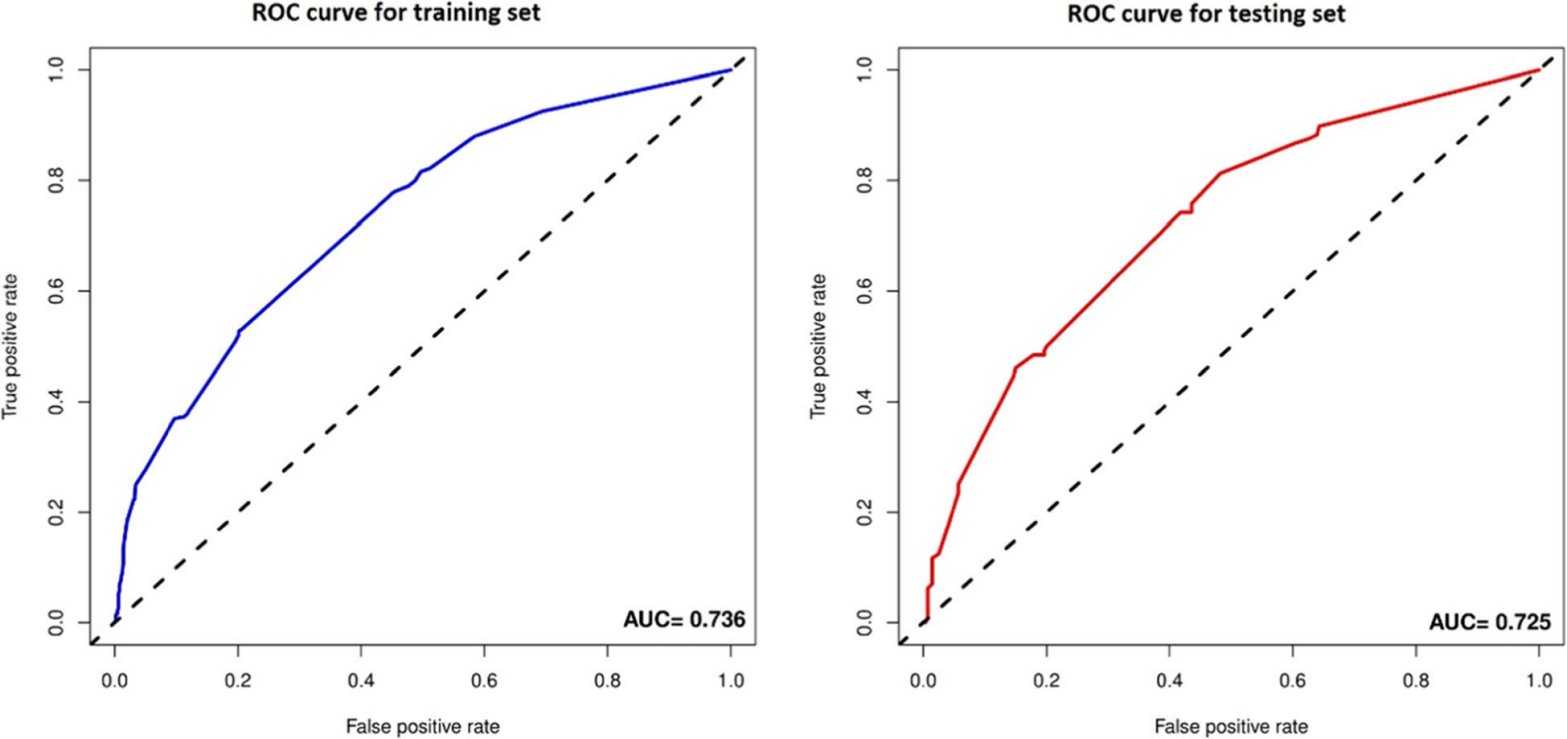
ROC curve of the nomogram. Notes: The ROC curves of the training set and testing set. The AUC of the training set is 0.736 and 0.725 in the testing set

**Fig. 4:**
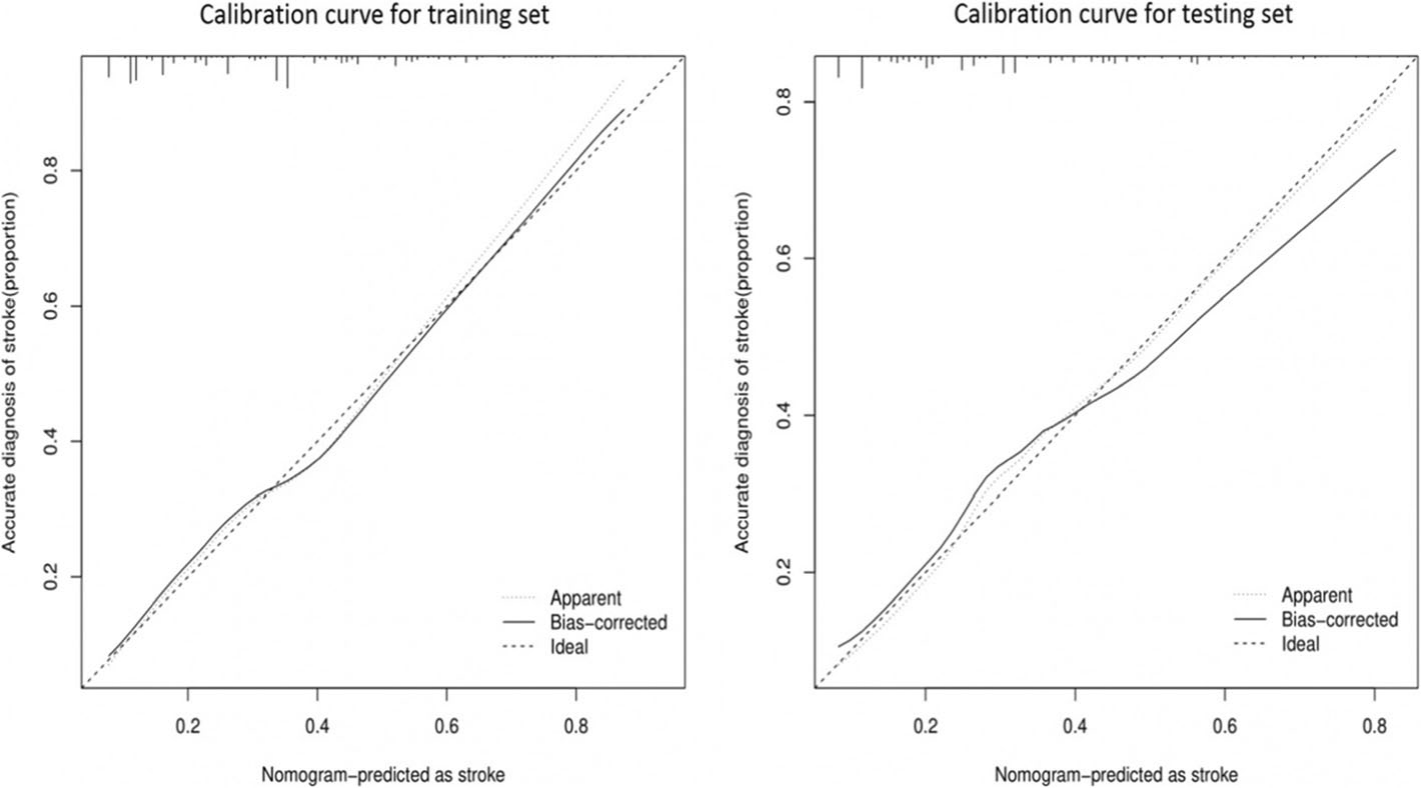
Calibration curve of the nomogram. **Notes:** The x-axis represents the risk predicted by the nomogram. The y-axis represents the patients diagnosed with acute ischemic stroke. The diagonal dotted line represents a perfect prediction by an ideal model. The apparent line represents the performance of the nomogram

The AUC of the prediction model was 0.736 in the training set, while the AUC after 1000-times bootstrap resampling was 0.730 and 0.725 in the external verification using the testing set (Fig. 3). The calibration curve illustrated an overlap between the probabilities of the predicted and actual diagnosis of stroke in both the training set and the testing set (Fig. 4).

### Multivariable logistic regression analysis and machine learning

**Fig. 5:**
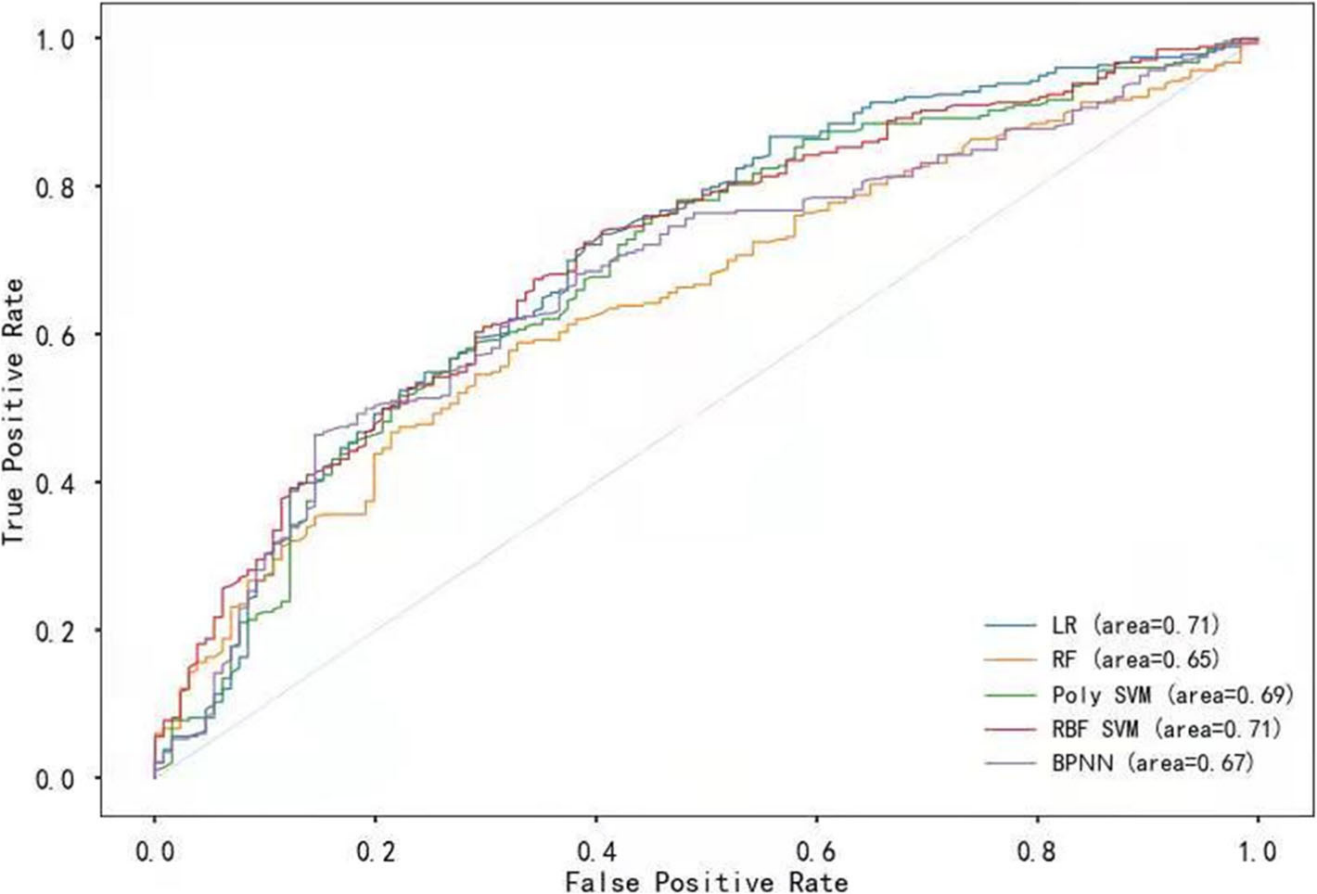
ROC curve of the machine learning and multivariable logistic regression. **Notes:** LR = logistic regression, RF = random forest, Poly SVM = support vector machine with polynomial kernel, RBF SVM = support vector machine with radial basis function kernel, BPNN = backpropagation neural network

We constructed the prediction model based on the same variables using the five different algorithms, and verified them using the testing set. The multivariable logistic regression analysis and support vector machine with radial basis function kernel both achieved an AUC score of 0.71 that was better than the other three prediction models (Fig. 5).

## Discussion

This study developed a practical nomogram that includes six variables that can be easily identified in the clinic to assist physicians in discriminating patients with high risk of stroke, enabling them to implement preventive measures as early as possible.

Blood pressure management is the most important variable that has a positive effect on stroke. With aging, the vascular elasticity decreases as a consequence of atherosclerosis. Thus, it is recommended that the systolic blood pressure in the elderly is less than 150 mmHg [15]. A meta-analysis reported that there was a 41% reduction in stroke for every blood pressure reduction of 10 mmHg systolic or 5 mmHg diastolic [16]. Although various hypertension guidelines indicate a certain goal of blood pressure control, few large-scale clinical evidence-based data focus on hypertension or stroke in very elderly patients. Professional doctors should be aware of this practical clinical problem and pay attention to the notion of individualized blood pressure management in elderly patients [17], without ignoring the symptoms and feelings of very elderly patients. In addition to the absolute value of blood pressure, blood pressure variability deserves attention. Excessive blood pressure fluctuation in the morning is a classic phenomenon. Kario used ambulatory blood pressure monitoring and magnetic resonance imaging and demonstrated that an exaggerated early morning blood pressure surge was independently associated with stroke in elderly hypertensive patients. The risk of stroke in patients with a morning blood pressure surge > 55 mmHg was 2.7 times higher than that in patients with a morning blood pressure surge < 55 mmHg. Pierdominico reached a similar conclusion that stroke had a relationship with an exaggerated early morning blood pressure surge independent of the 24-h average blood pressure [18, 19].

Smoking and alcoholism are controllable risk factors for stroke. Both played an important role in our prediction model, and these were valid for more than 90% of the males in our cohort. A large number of clinical studies in different races and populations have confirmed the strong association between smoking and stroke, while exposure to secondhand smoke should also be noted. Current smokers are at least two-to-four times more likely to have a stroke than those who never smoked or those who quit smoking 10 years ago [20]. Some epidemiological studies have demonstrated that the impact of drinking on stroke risk depends on the quantity. A small amount of red wine may reduce the risk of cardiovascular disease and stroke. However, alcohol abuse (> 60 g/day) is associated with an increased risk of stroke in the long term [21, 22].

CAS is a marker of systemic atherosclerosis that can be easily detected by ultrasound. According to studies from the 1980s, the annual risk of ipsilateral stroke was 3% in patients with a CAS ≥ 50%, which increased to 5.5% in patients with a CAS > 75%. With the widespread use of preventive drugs, the annual risk of stroke has been reduced to 0.34% for patients with a CAS ≥ 50% in contemporary studies [23, 24].

Other risk factors that are not included in our nomogram, such as age, total cholesterol and LDL-cholesterol [25–27], were proven to be related to stroke by an abundance of clinical trials and should be considered by clinicians. It is worth noting that elderly patients usually present with multiple chronic diseases, such as hypertension, diabetes and coronary heart disease. The risk of ischemic stroke caused by pathological changes of organs caused by these diseases may be more serious than that caused by physiological aging [28]. Additionally, elderly patients often do not adhere to prescribed treatments. The direct visual display of the nomogram model can play a role in educating elderly patients and increase their compliance to treatment.

In the era of artificial intelligence, machine learning has become a popular method in data analysis. It utilizes mathematical models and training data to make predictions [29, 30]. The random forest, support vector machines, and backpropagation neural networks are three representative algorithms of machine learning that are increasingly used in the prediction of adverse events in clinical practice or biological research in tumor [31, 32]. Although these machine learning algorithms have attracted much attention with the availability of increasingly voluminous datasets (such as electronic medical records), the internal process of which is similar to a “black box” with poor interpretability and visualization, limit their practical application.

In a number of reports, the results of multivariable logistic regression analysis as the classic reference standard were compared with those of machine learning algorithms. In our study, the machine learning algorithms offered no obvious advantage over multivariable logistic regression in evaluating a binary categorical problem (whether or not patients will suffer an acute ischemic stroke). This conclusion is the same as that of several recent studies [14, 33].

Our prediction model based on multivariable logistic regression analysis not only has considerable accuracy but also can be visualized by a nomogram, which is convenient for its clinical application.

### Limitations

This study was a single-center retrospective study, which limits its generalizability. As a retrospective study, potential selection bias was inevitable. Furthermore, there are numerous other stroke-related risk factors, such as the body mass index, diet habits, and physical exercise, that were not analyzed because they were not reported in the electronic records of patients.

## Data Availability

The datasets used and/or analyzed during the current study are available from the corresponding author on reasonable request.

## Abbreviations

TC: Total cholesterol
LDL-C: Low density lipoprotein cholesterol
HDL-C: High density lipoprotein cholesterol
OR: Odds ratio
CI: Confidence interval
ROC: Receiver operating characteristic curve
AUC: Area under the curve
RF: Random forest
SVM: Support vector machine
BPNN: Back propagation neural network
